# Nuclear-Cytoplasmic Trafficking of NTF2, the Nuclear Import Receptor for the RanGTPase, Is Subjected to Regulation

**DOI:** 10.1371/journal.pone.0042501

**Published:** 2012-08-03

**Authors:** Shawn C. Chafe, Jacqueline B. Pierce, Dev Mangroo

**Affiliations:** Department of Molecular and Cellular Biology, University of Guelph, Guelph, Ontario, Canada; McGill University, Canada

## Abstract

NTF2 is a cytosolic protein responsible for nuclear import of Ran, a small Ras-like GTPase involved in a number of critical cellular processes, including cell cycle regulation, chromatin organization during mitosis, reformation of the nuclear envelope following mitosis, and controlling the directionality of nucleocytoplasmic transport. Herein, we provide evidence for the first time that translocation of the mammalian NTF2 from the nucleus to the cytoplasm to collect Ran in the GDP form is subjected to regulation. Treatment of mammalian cells with polysorbitan monolaurate was found to inhibit nuclear export of tRNA and proteins, which are processes dependent on RanGTP in the nucleus, but not nuclear import of proteins. Inhibition of the export processes by polysorbitan monolaurate is specific and reversible, and is caused by accumulation of Ran in the cytoplasm because of a block in translocation of NTF2 to the cytoplasm. Nuclear import of Ran and the nuclear export processes are restored in polysorbitan monolaurate treated cells overproducing NTF2. Moreover, increased phosphorylation of a phospho-tyrosine protein and several phospho-threonine proteins was observed in polysorbitan monolaurate treated cells. Collectively, these findings suggest that nucleocytoplasmic translocation of NTF2 is regulated in mammalian cells, and may involve a tyrosine and/or threonine kinase-dependent signal transduction mechanism(s).

## Introduction

Eukaryotic cells compartmentalize the DNA replication and transcription apparatus in the nucleus and the translation machinery in the cytoplasm. This segregation requires that exchange of molecules between the two compartments takes place across the double lipid bilayer of the nuclear envelope in order for both processes to function optimally. The nuclear envelope is perforated with large proteinaceous assemblies known as nuclear pore complexes (NPCs). These macromolecular complexes range in size from 50 MDa in yeast to 125 MDa in vertebrates [1]. The protein components comprising the NPC belong to a group of proteins called nucleoporins (Nups). The central channel of the NPC is lined with a population of Nups containing multiple FG dipeptide repeats, which are thought to provide a hydrophobic barrier that serves to control passage through the pore [2]. The inner dimensions of the pore govern the size of macromolecules allowed to freely diffuse through the channel. The passage of ions and molecules less than 60 kDa in size through the pore occurs by simple diffusion. However, some proteins and RNAs that are smaller than the 60 kDa exclusion limit are not free to diffuse across the pore even though they are below the size restriction of the inner core; these molecules and those that are much larger in size require a carrier-mediated active transport process in order to move through the NPC.

Nucleocytoplasmic trafficking of macromolecules is controlled by proteins that have the ability to move freely through the pore of the NPC. The proteins mediating the exchange are known as nuclear transport receptors (NTRs). NTRs are able to identify and bind to targeting signals within the cargo dictating whether the cargo will end up in the nucleus or the cytoplasm. Proteins that are destined to the nucleus possess a nuclear localization signal (NLS), and proteins targeted for the cytoplasm contain a nuclear export signal (NES). The best characterized pathway for the exchange of molecules between the nucleus and the cytoplasm is by a family of NTRs that resemble Importin-β. This family of proteins is known as β-karyopherins and consists of more than 20 known members in metazoans (for review, see [3]). β-karyopherins are further divided into importins and exportins based on their function. For import, the best characterized example is that of import of cargoes possessing the classical lysine-rich NLS by Importin-α. Importin-α binds the NLS bearing protein in the cytoplasm, and this complex is then bound by Importin-β; the trimeric complex associates with, and translocates through the NPC [4], [5]. Upon reaching the nucleoplasmic side of the nucleus, the import complex is dissociated by binding of RanGTP to Importin-β. Importin-α is then returned to the cytoplasm for another round of import by the RanGTP-binding protein CAS [6], [7]. Protein export occurs by a similar mechanism, requiring the recognition of the NES containing cargo by the exportin such as Crm1 in the nucleus. However, exportin binding to the cargo is dependent on interaction with RanGTP. The export complex consisting of exportin-cargo-RanGTP exits the nucleus through the NPC [8], and upon reaching the cytoplasm, the GTPase activity of Ran is activated. Hydrolysis of GTP to GDP by Ran causes the export complex to dissociate. Some RNAs such as tRNAs, are also exported out of the nucleus by a β-karyopherin. In addition, export of these RNAs from the nucleus by β-karyopherins, such as the nuclear tRNA export protein exportin-t (Xpo-t) is dependent on RanGTP [9], [10].

Ran is a small Ras-like GTPase that is involved in a number of cellular processes which include: cell cycle regulation, chromatin organization during mitosis, reformation of the nuclear envelope following mitosis and controlling the directionality of nucleocytoplasmic transport (for review, see [11]). Like other members of the Ras super family, Ran acts as a switch to control nucleocytoplasmic transport as it changes between its GTP and GDP bound nucleotide states. The nucleotide exchange factor for Ran is RCC1. RCC1 is chromatin bound and is responsible for exchanging the GDP bound to Ran for GTP, thereby providing the nucleus with an abundance of RanGTP [12]. Ran has very little intrinsic GTPase activity and therefore requires further activation by RanGAP (RanGTPase activating protein) [13]. RanGAP is sumoylated and tethered to the cytoplasmic face of the NPC by its association with RanBP2/Nup358 [14], [15]. The spatial organization of the components that control the nucleotide state of Ran creates a RanGTP gradient across the NPC. This gradient is responsible for controlling the directionality of transport, as import complexes dissociate in the presence of RanGTP and export complexes require RanGTP for proper formation and hydrolysis of GTP by Ran for dissociation [12], [16]. Therefore, Ran exits the nucleus in the GTP bound form by its association with the export complex and returns to the nucleus in the GDP bound form. NTF2 was first identified by its ability to stimulate nuclear import in permeabilized cells [17], [18]. Subsequently, it was shown to be responsible for RanGDP import into the nucleus [19], [20]. NTF2 binds directly to RanGDP and once bound it is able to interact more efficiently with the NPC allowing for the complex to translocate into the nucleus [19], [21]. Once in the nucleoplasm the mechanism of RanGDP dissociation from NTF2 is unclear, but it is believed to be due to the increased concentration of GTP encountered, for which NTF2 has no affinity [19], [22].

The importance of proper regulation of nucleocytoplasmic exchange of molecules is well documented [23], [24], and the intracellular signaling pathways responsible for controlling the regulation of nucleocytoplasmic transport are slowly being identified [25]. Numerous cellular stresses have led to a reorganization of the machinery required for transport through the NPC. In mammals, hyperosmotic stress implicated the p38 MAPK cascade [26], and a block in the phosphorylation of RanBP3 implicated the PI3K pathway [27] in controlling the cellular distribution of Ran, whereas oxidative stress has implicated the ERK1/2 and Akt pathways [28] in controlling the location of various members of the β-karyopherin family. In *Saccharomyces cerevisiae* nitrogen or amino acid starvation also causes nuclear retention of tRNAs made from intron-containing pre-tRNAs [29]–[31]. Moreover, inhibition of TORC1, which is normally turned off under low nitrogen conditions, by treatment of *S. cerevisiae* with rapamycin leads to inhibition of nuclear export of mature tRNAs made from intron-containing pre-tRNAs. These findings indicate that the TORC1 signaling pathway controls nuclear tRNA export in response to nitrogen availability [31]. However, it is presently not known whether mTOR also controls nuclear tRNA export in mammals. Herein, we investigated this possibility and show that mTORC1 is not involved in the regulation of nuclear tRNA export in mammalian cells. However, during this investigation we found that polysorbitan monolaurate (Tween-20), a component of the solution used to deliver rapamycin to the cells, causes cytoplasmic accumulation of Ran by blocking translocation of NTF2 to the cytoplasm. This disruption had serious consequences on numerous nucleocytoplasmic export processes that are dependent on RanGTP function in the nucleus, but none on nuclear import of proteins. These effects of Tween-20 are reversible and specific, as other detergents had no effect on the nuclear-cytoplasmic export processes. The block in translocation of NTF2 from the nucleus to the cytoplasm is not related to Tween-20 causing the cells to undergo apoptosis. However, inhibition of nuclear import of Ran and the nuclear export processes by prolonged Tween-20 treatment led to apoptosis. Moreover, overexpression of NTF2 restored nuclear import of Ran and nuclear export processes in Tween-20 treated HeLa cells. Tween-20 treatment was found to cause an increase in the level of phosphorylation of some phospho-tyrosine and phospho-threonine proteins. However, NTF2 was not phosphorylated in response to Tween-20 treatment. These data suggest that Tween-20 is affecting a signal transduction mechanism that is responsible for regulating nuclear-cytoplasmic translocation of NTF2. This is the first demonstration that cytoplasmic localization of NTF2 in mammalian cells is regulated, and may involve a tyrosine and/or threonine kinase-dependent signaling pathway(s). Moreover, we anticipate that Tween-20 can be used as a tool to identify the mechanism(s) regulating translocation of NTF2 from the nucleus to the cytoplasm.

## Results

### Tween-20 but not rapamcyin causes a block in nuclear tRNA export in mammalian cells

The mammalian target of rapamycin (mTOR) has been shown to play a pivotal role in the regulation of a number of critical cellular processes, including translation, ribosome biogenesis, and transcription of tRNA genes [32]. Inhibition of TORC1 function with the drug rapamycin, which simulates nitrogen deprivation, leads to an arrest in the progression of the cell cycle [32]. Treatment of *S. cerevisiae* with rapamycin, as well as nitrogen or amino acid deprivation has been shown to cause nuclear accumulation of mature tRNAs derived from intron-containing precursors, but not those made from intronless precursors [30], [31]. These data demonstrated that a regulatory mechanism in *S. cerevisiae* controls nuclear export of mature spliced tRNAs in response to amino acid or nitrogen availability and that TORC1, in part, plays a role in this regulation [31]. Recent studies have also demonstrated that tRNAs accumulate in the nucleus of rat hepatoma H4IIE cells during amino acid deprivation [33], suggesting that mammals may also possess a regulatory mechanism to control nuclear tRNA export during nutrient stress. Consequently, we investigated whether mTORC1 may be involved in regulating nuclear tRNA export in mammalian cells.

**Figure 1:**
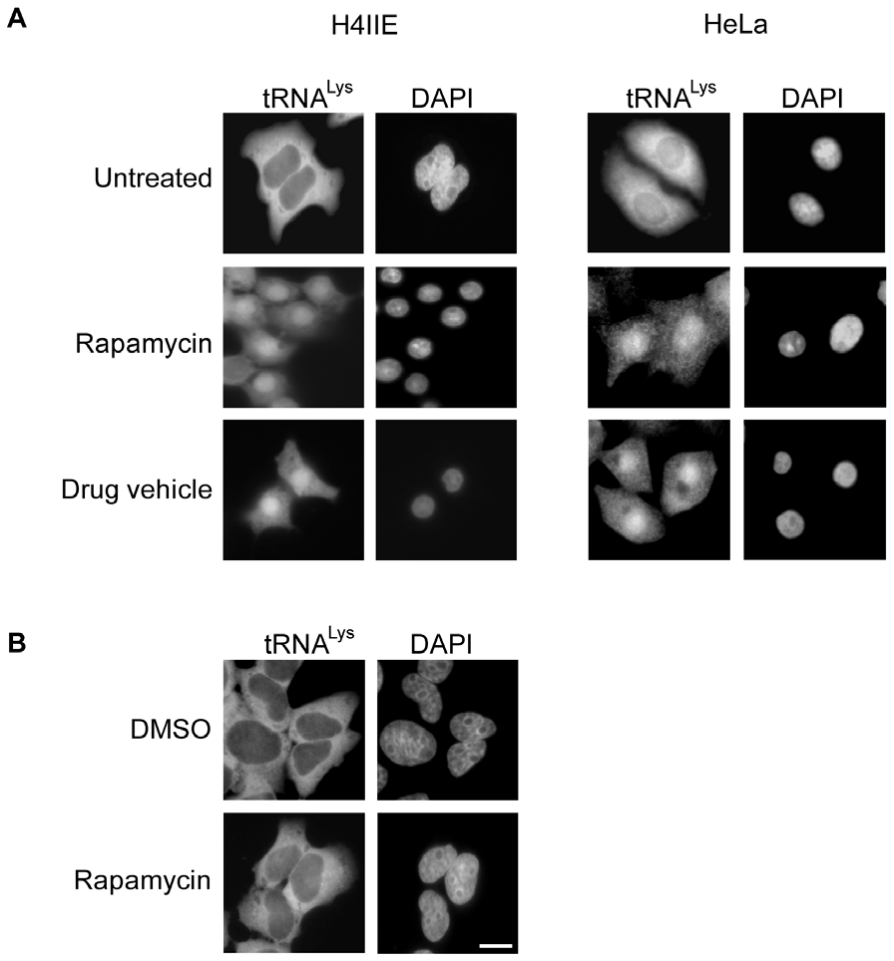
Rapamycin treatment does not affect nuclear tRNA export. (A) Rat hepatoma H4IIE and HeLa cells were treated with 100 nM rapamycin or the drug vehicle (DV) solution composed of 90% ethanol (v/v) and 10% Tween-20 (v/v) for 4 h in serum-free DMEM. (B) The same cell lines were treated with 100 nM rapamycin for 4 h using DMSO as a drug vehicle. The treated cells were fixed in 1× PBS containing 4% formaldehyde, and the distribution of tRNA^Lys^ was monitored by FISH. The cells were stained with DAPI to delineate the nucleus. Scale bar represents 10 µm.

To ascertain whether TORC1 was involved in controlling nuclear tRNA export in mammals, FISH was used to monitor the cellular distribution of tRNA^Lys^ in HeLa and rat hepatoma H4IIE cells incubated in DMEM without serum and containing the drug vehicle solution (see figure legend) with or without 100 nM rapamycin for 4 h (Figure 1A). An increased nuclear signal for tRNA^Lys^ was observed for both cell lines treated with rapamycin and with the drug vehicle solution alone. In contrast, tRNA^Lys^ was not detected in the nucleus of untreated cells. This surprising finding suggests that a component of the drug vehicle solution, and not rapamycin, was responsible for the defect observed in nuclear tRNA export.

To verify that rapamycin was not affecting nuclear tRNA export in mammals, the location of tRNA^Lys^ was monitored in rat hepatoma H4IIE and HeLa cells treated for 4 h with rapamycin solubilized in DMSO (Figure 1B). The distribution of tRNA^Lys^ in cells treated with 100 nM rapamycin in DMSO or DMSO alone was mostly cytoplasmic. Similarly, treatment with rapamycin or DMSO did not affect localization of tRNA^Leu^ to the cytoplasm in HeLa cells (data not shown). These data confirm that the nuclear tRNA export defect observed is unrelated to rapamycin, and suggest that TORC1 may not play a role in regulating nuclear tRNA export in mammalian cells. Instead, the data suggest that the ethanol or Tween-20 component of the drug vehicle solution may be responsible for causing the observed nuclear tRNA export defect.

**Figure 2:**
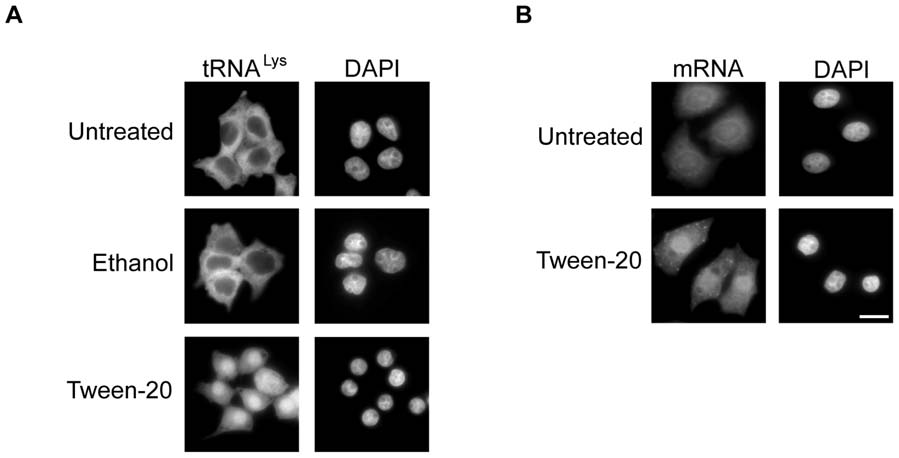
Tween-20 causes a defect in nuclear tRNA and mRNA export. (A) Rat hepatoma H4IIE cells were treated with 0.16% ethanol, the concentration of ethanol the cells received during drug vehicle treatment, or 150 µM Tween-20 in serum-free DMEM for 4 h. The cells were fixed and the distribution of the tRNA was monitored by FISH. (B) HeLa cells were treated with 150 µM Tween-20 for 4 h in serum-free DMEM, fixed in 1× PBS containing 4% formaldehyde and FISH was used to monitor the distribution of mRNA. The cells were stained with DAPI to delineate the nucleus. Scale bar represents 10 µm.

To decipher which of the two components of the drug vehicle was responsible for the defect in nuclear tRNA export, rat hepatoma H4IIE cells were treated with ethanol or Tween-20 for 4 h in serum-free DMEM and processed for FISH to monitor the cellular location of tRNA^Lys^ (Figure 2A). In cells treated with ethanol, tRNA^Lys^ was found exclusively in the cytoplasm (middle row). A similar distribution was observed for tRNA^Lys^ in untreated cells (top row). However, the cells treated with the same concentration of Tween-20 (150 µM, critical micellar concentration (CMC) of Tween-20 is 80 µM) present in the drug vehicle solution displayed a defect in nuclear tRNA export similar to those cells treated with the drug vehicle solution (bottom row). This data clearly indicate that Tween-20 was affecting nuclear tRNA export.

To determine whether the observed effect was specific to nuclear tRNA transport, and not a general effect on all nuclear RNA transport processes, HeLa cells were incubated in serum-free DMEM with or without 150 µM Tween-20 for 4 h and the distribution of mRNA was monitored by FISH (Figure 2B). The location of mRNA was detected with an AlexaFluor-488-oligo-dT oligonucleotide. The results show that mRNA was uniformly distributed in untreated cells. However, in Tween-20 treated cells, mRNA was located primarily in the nucleus (bottom panel), suggesting that Tween-20 may have a general effect on nuclear export processes. This conclusion is supported by findings described below.

### Tween-20 but not Tween-80 or deoxycholate affects nuclear tRNA transport

**Figure 3:**
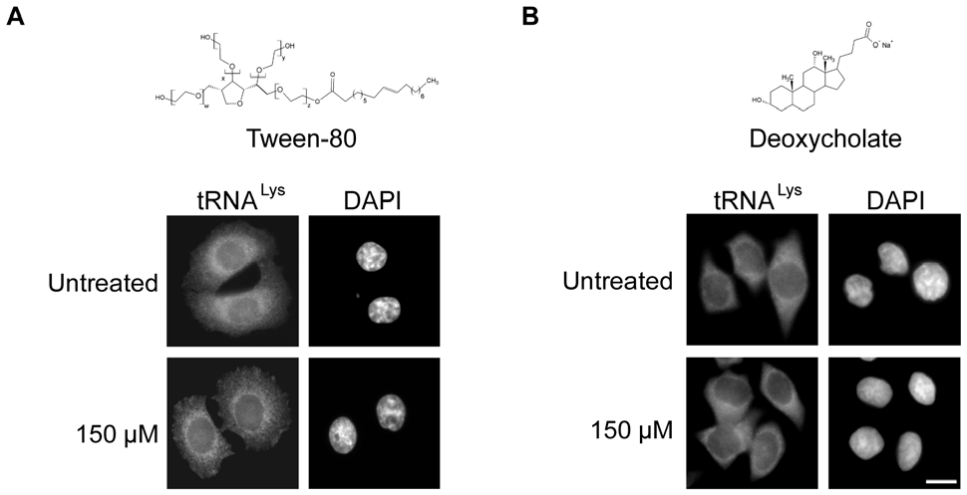
Tween-80 or deoxycholate treatment does not affect nuclear tRNA export. HeLa cells were treated with (A) 150 µM Tween-80 or (B) 150 µM deoxycholate for 4 h in serum-free DMEM. The cells were fixed and FISH was used to monitor the distribution of tRNA^Lys^. The cells were stained with DAPI to visualize the nucleus. Scale bar represents 10 µm.

Tween-20 is an amphipathic detergent composed of a sorbitol head group attached to 4 polyoxyethylene groups and one lauric acid moiety. It differs from other members of the Tween family based on the length of the polyoxyethylene chain and the fatty acid moiety attached to the head group. To test whether the effect observed on nuclear tRNA and mRNA export was specific to Tween-20, or could be caused by another member of the Tween family that possesses a longer fatty acid chain, the effect of Tween-80 on nuclear tRNA export in HeLa cells was investigated (Figure 3A). Tween-80 has an oleic acid (C-18) unsaturated chain attached to the head group, which differs from the shorter saturated lauric acid (C-12) chain of Tween-20. HeLa cells were incubated in serum-free DMEM without or with 150 µM Tween-80 (CMC is 12 µM) for 4 h and the distribution of tRNA^Lys^ was monitored by FISH. The cells maintained in DMEM alone displayed cytoplasmic distribution of tRNA^Lys^. Furthermore, there was no change in the distribution of tRNA^Lys^ in cells treated with Tween-80, suggesting that the fatty acid chain length and saturation may be an important part of the Tween molecule's ability to affect nuclear tRNA export.

To test whether a detergent outside of the Tween family could block nuclear tRNA export, HeLa cells were incubated in serum-free DMEM without or with 150 µM deoxycholate (CMC is 6 mM) for 4 h (Figure 3B). As observed before, tRNA^Lys^ was found in the cytoplasm in untreated cells. Like Tween-80, deoxycholate also did not affect nuclear export of tRNA^Lys^. These data taken together suggest that the effect observed on nuclear tRNA and mRNA transport may be specific to the detergent Tween-20.

### The entire Tween-20 molecule is required to block nuclear tRNA export in HeLa cells

**Figure 4:**
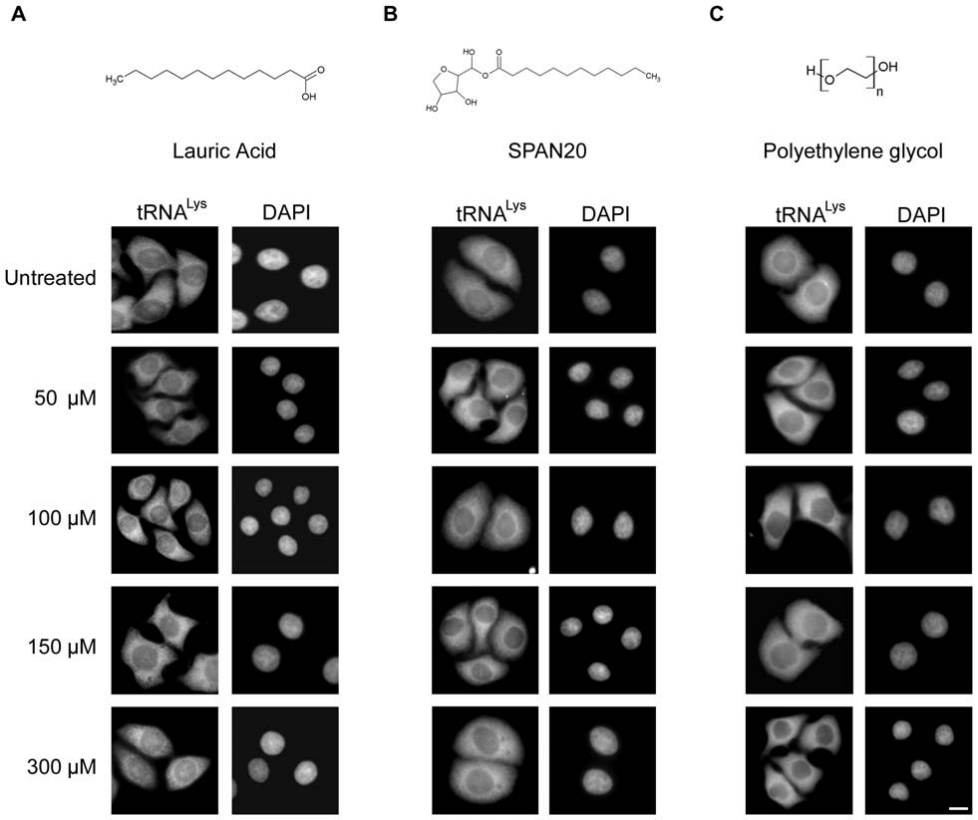
The entire Tween-20 molecule is required to block nuclear tRNA export in HeLa cells. HeLa cells were treated with 50–300 µM (A) lauric acid (B), SPAN20 and (C) polyethylene glycol for 4 h in serum-free DMEM. The cells were fixed and FISH was used to monitor the location of tRNA^Lys^. The cells were stained with DAPI to visualize the nucleus. Scale bar represents 10 µm.

To determine which portion of the Tween-20 molecule was required to block nuclear tRNA export, HeLa cells were treated with each individual constituent (Figure 4). The cells were incubated with 50–300 µM lauric acid for 4 h in serum-free DMEM or in serum-free DMEM alone, and the cellular distribution of tRNA^Lys^ was monitored by FISH. tRNA^Lys^ was found mainly in the cytoplasm of untreated cells (Figure 4A) and in cells treated with various concentrations of lauric acid. Therefore, although the fatty acid chain length may be important for the action of Tween-20 on these cells, the lauric acid moiety alone is not sufficient to block nuclear tRNA export. Similarly, SPAN20 (sorbitan monolaurate), which is the Tween-20 molecule lacking the polyoxyethylene chains has no effect on nuclear tRNA export, irrespective of the concentration of SPAN20 used (Figure 4B). In addition, the sorbitol moiety has no effect on nuclear tRNA export (data not shown). The polyethylene groups also did not affect nuclear tRNA export, as tRNA^Lys^ was found in the cytoplasm of cells incubated with 300 µM polyethylene glycol (PEG) (Figure 4C). Taken together, the data suggest that the entire Tween-20 molecule may be required to block nuclear RNA export in HeLa cells.

### The defect in RNA export caused by Tween-20 is reversible

**Figure 5:**
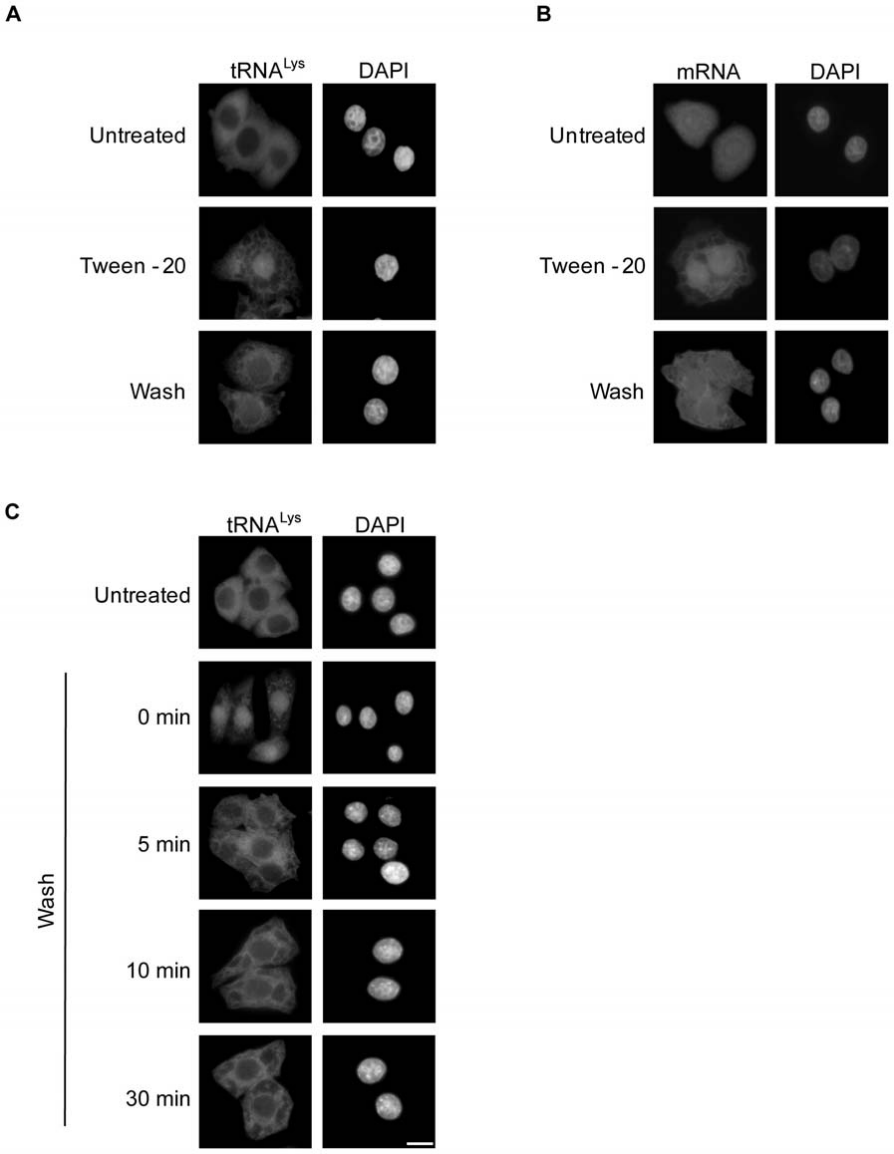
The nuclear tRNA and mRNA export defect caused by Tween-20 is reversible. HeLa cells were left in serum-free media (panels A and B, Untreated) or treated with 150 µM Tween-20 for 4 h in serum-free DMEM. The cells were then kept in the presence of Tween-20 (second panel, Tween-20), or washed and placed in fresh serum-free DMEM for 1 h (third panel, Wash). The cells were then fixed and processed for FISH to monitor the distribution of (A) tRNA^Lys^ or (B) mRNA. (C) Tween-20 treated cells were washed and placed in serum-free DMEM and incubated for the times indicated. The location of tRNA^Lys^ was monitored by FISH. The cells were DAPI stained to visualize the nucleus. Scale bar represents 10 µm.

To understand how Tween-20 is affecting tRNA and mRNA export, we investigated whether Tween-20 induced nuclear retention of the RNAs could be reversed (Figure 5). HeLa cells were incubated in serum-free DMEM or serum-free DMEM containing 150 µM Tween-20 for 4 h. The cells were either maintained in the same medium containing Tween-20, or washed and placed in fresh serum-free medium and incubated for 1 h. Cells that were incubated in medium without Tween-20 showed cytoplasmic distribution of tRNA^Lys^ (Figure 5A) and normal distribution of mRNA (Figure 5B). In agreement with previous results, cells treated with Tween-20 retained both tRNA^Lys^ (Figure 5A) and mRNA (Figure 5B) in the nucleus. Interestingly, tRNA^Lys^ and mRNA redistribute to the cytoplasm in Tween-20 treated cells that were washed and placed in fresh serum-free medium lacking Tween-20 (Figure 5A and B). The restoration of nuclear tRNA export was also observed as early as 5 min after the Tween-20 treated cells were incubated in Tween-20-free medium (Figure 5C). These data indicate that the nuclear tRNA and mRNA export defects caused by Tween-20 treatment could be reversed by removal of the Tween-20 stimulus. Moreover, these findings demonstrate that the effect of Tween-20 on these two essential transport processes in HeLa cells is reversible and that the cells have the capacity to recover upon removal of the external stimulant. Thus, it is unlikely that Tween-20 is causing major physical damage to the cells or covalently modifying an important component that influences nuclear tRNA and mRNA export. This interpretation is consistent with data showing that Tween-20 treated HeLa cells, like untreated cells, exclude Trypan Blue, a dye that is used to monitor the integrity of the plasma membrane of mammalian cells (data not shown).

### Tween-20 does not affect nuclear localization of Xpo-t

**Figure 6:**
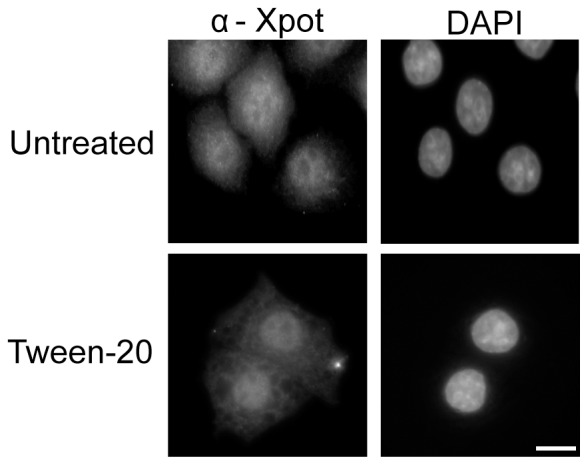
Tween-20 does not affect nuclear localization of Xpo-t in HeLa cells. HeLa cells were incubated in fresh serum-free DMEM (Untreated) without or with 150 µM Tween-20 for 4 h (Tween-20). The cells were washed and fixed with 4% paraformaldehyde in 1× PBS and the distribution of Xpo-t was monitored by immunofluorescence microscopy. The cells were stained with DAPI to visualize the nucleus. Scale bar represents 10 µm.

Studies reported have demonstrated that cellular stress causes an adjustment of the function of nuclear transport receptors as cells adapt to the insult [28], [34]–[37]. This readjustment typically involves a change in the localization of the transport receptors, or the efficiency of translocation through the NPC [28], [34]–[37]. To understand how Tween-20 causes a block in RNA export, we investigated whether nuclear-cytoplasmic trafficking of Xpo-t, which is the main β-karyopherin required for nuclear tRNA export in mammalian cells, was affected [9], [38]. Los1p, the *S. cerevisiae* orthologue of Xpo-t, has been shown to be retained in the cytoplasm during DNA damage stress [37]. Therefore, we tested whether the cellular response triggered by Tween-20 leading to the observed tRNA export defect was caused by mislocalization of Xpo-t to the cytoplasm (Figure 6). In untreated HeLa cells, Xpo-t is located mainly in the nucleus. Furthermore, the localization of Xpo-t in cells treated with 150 µM Tween-20 for 4 h does not change and continued to show a distinct nuclear signal. Thus, it is unlikely that the nuclear RNA export defect caused by Tween-20 treatment is due to mislocalization of the export receptors.

### Tween-20 causes Ran to accumulate in the cytoplasm of HeLa cells and a block in nuclear export of proteins, but does not affect nuclear import of proteins

**Figure 7:**
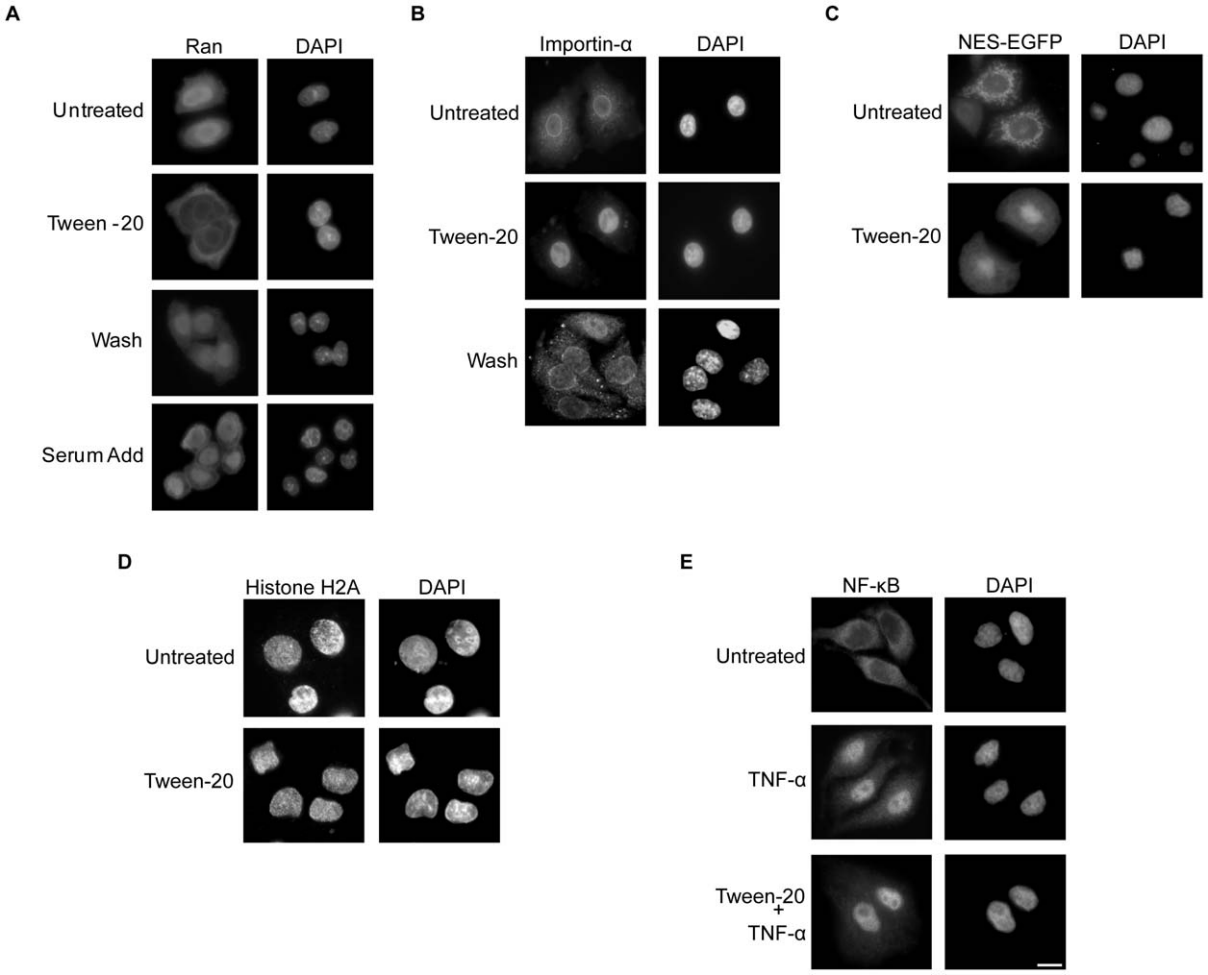
Tween-20 treatment causes Ran to accumulate in the cytoplasm of HeLa cells and a block in nuclear export of proteins but not nuclear import of proteins. (A) Tween-20 causes cytoplasmic retention of Ran in HeLa cells. HeLa cells were incubated in fresh serum-free DMEM (Untreated) or in serum-free DMEM containing 150 µM Tween-20 for 4 h. After the 4 h incubation, the cells were left in Tween-20 containing medium (Tween-20), washed and placed in fresh serum-free media (Wash), or had the serum-free DMEM media containing Tween-20 supplemented with 10% FBS (Serum Add) and incubated at 37°C for 1 h. The distribution of Ran was monitored by immunofluorescence microscopy as described in materials and methods. (B) Tween-20 causes nuclear accumulation of Importin-α. HeLa cells were incubated in serum-free DMEM without (Untreated) or with 150 µM Tween-20 in serum-free DMEM for 4 h (Tween-20). Following the 4 h incubation, the cells were washed and incubated in serum-free DMEM for 1 h. The distribution of Importin-α was monitored by immunofluorescence microscopy. (C) Tween-20 causes nuclear accumulation of an NES-EGFP. HeLa cells were transfected with NES-EGFP and allowed to express for 24 h. Post-transfection cells were washed and placed in fresh serum-free DMEM with (Tween-20) or without (Untreated) 150 µM Tween-20 for 4 h. The distribution of NES-EGFP was monitored by direct fluorescent microscopy. (D) Tween-20 does not block nuclear import of the NLS containing Histone H2A. HeLa cells were incubated in serum-free DMEM without (Untreated) or with 150 µM Tween-20 for 4 h. The distribution of Histone H2A was monitored by immunofluorescence microscopy. (E) Tween-20 does not affect TNF-α stimulated nuclear import of NF-κB. HeLa cells were incubated in serum-free DMEM without (Untreated) or with 10 ng/ml TNF-α for 30 min, or with 150 µM Tween-20 for 4 h and then for 30 min with 10 ng/ml TNF-α. The distribution of NF-κB was monitored by immunofluorescence microscopy. The cells were DAPI stained to visualize the nucleus. Scale bar represents 10 µm.

The small GTPase Ran in the GTP bound state has been shown to be required for nuclear tRNA export, as it facilitates loading of Xpo-t with the tRNA cargo [12]. Furthermore, β-karyopherins involved in nuclear import/export are also dependent on the function of RanGTP in the nucleus. A number of cellular insults have previously been reported to cause a change in the localization of Ran [26], [39]. Since mislocalization of Ran may be responsible for the block in nuclear tRNA export, we investigated whether the distribution of Ran was altered in cells treated with Tween-20. HeLa cells were incubated in serum-free DMEM or in serum-free DMEM containing 150 µM Tween-20 for 4 h. After the incubation the cells were left in Tween-20 containing medium (Tween-20), washed and placed in fresh serum-free DMEM (Wash), or the Tween-20- containing media was supplemented with serum to a final concentration of 10% (Serum add) and incubated for 1 h (Figure 7A). Immunofluorescence microscopy indicates that Ran is located primarily in the nucleus in HeLa cells incubated in complete DMEM medium [26], [39] (data not shown), and the nuclear location of Ran did not change when HeLa cells were incubated in serum-free DMEM lacking Tween-20. In contrast, Ran was found primarily in the cytoplasm of Tween-20 treated cells. Furthermore, Ran re-localizes to the nucleus when Tween-20 containing DMEM was replaced with Tween-20-free DMEM, or when the Tween-20-containing media was supplemented with serum. These data show that Tween-20 causes a block in nuclear import of Ran, and suggest that the loss of Ran from the nucleus is responsible for the nuclear mRNA and tRNA export defects observed.

To ascertain whether mislocalization of Ran has an impact on other Ran-dependent nuclear import/export processes, we investigated the location of Importin-α to monitor classical protein import (Figure 7B) and an NES-EGFP chimera to monitor Crm1-mediated nuclear protein export (Figure 7C). Immunofluorescence microscopy demonstrated that in untreated HeLa cells Importin-α was primarily in the cytoplasm and at the nuclear periphery (Figure 7B, top), which is consistent with results reported previously [5], [34]. However, in cells treated with 150 µM Tween-20 for 4 h in serum-free DMEM, Importin-α was located in the nucleus. This suggests that while import of Importin-α to the nucleus was not affected by Tween-20 treatment, the return of the protein to the cytoplasm, which is known to be a Ran-dependent process, was blocked [6] (Figure 7B). Furthermore, nuclear export of Importin-α was restored by removing Tween-20 (Figure 7B). When NES-EGFP was transfected into HeLa cells and allowed to express for 24 h it was observed mainly in the cytoplasm and at the nuclear periphery, which is also consistent with data from previous studies [40] (Figure 7C). However, upon Tween-20 treatment for 4 h in serum-free DMEM medium the EGFP signal was found to accumulate in the nucleus (Figure 7C, bottom), suggesting that nuclear export of proteins by Crm1 was also affected. Moreover, consistent with the restoration of Ran and Importin-α to their proper location, the export of the NES-EGFP chimera was restored upon removal of Tween-20 (data not shown).

To test more directly whether nuclear import of proteins was affected, HeLa cells were treated with Tween-20 and the import of Histone H2A, which is an NLS bearing protein, was monitored (Figure 7D). Histone H2A was found entirely in the nucleus in untreated cells, which is consistent with previous studies [41]. Histone H2A was also found exclusively in the nucleus in Tween-20 treated cells. In addition, no detectable Histone H2A signal was observed in the cytoplasm of Tween-20 treated cells, suggesting that nuclear import of Histone H2A was not affected. This data also indicates that nuclear import of NLS containing proteins is not affected during Tween-20 treatment.

NF-κB has been shown to translocate to the nucleus of cells stimulated with TNF-α [42]. Thus, the effect of Tween-20 treatment on nuclear import of NF-κB in HeLa cells was also investigated (Figure 7E). In agreement with previous studies, NF-κB is found primarily in the cytoplasm of untreated cells and in the nucleus upon stimulation with TNF-α. NF-κB also locates to the nucleus of Tween-20 treated HeLa cells stimulated with TNF-α, indicating that Tween-20 does not affect nuclear protein import processes. Taken together, the data suggest that retention of Ran in the cytoplasm not only affects nuclear tRNA and mRNA export, but has caused a global effect on nuclear export processes requiring the activity of Ran in the nucleus. Importantly, these data also suggest that nuclear import processes are not affected by Tween-20 treatment.

### Tween-20 does not appear to affect localization of RanGAP to the NPC, or the activities of Ran and RanGAP

**Figure 8:**
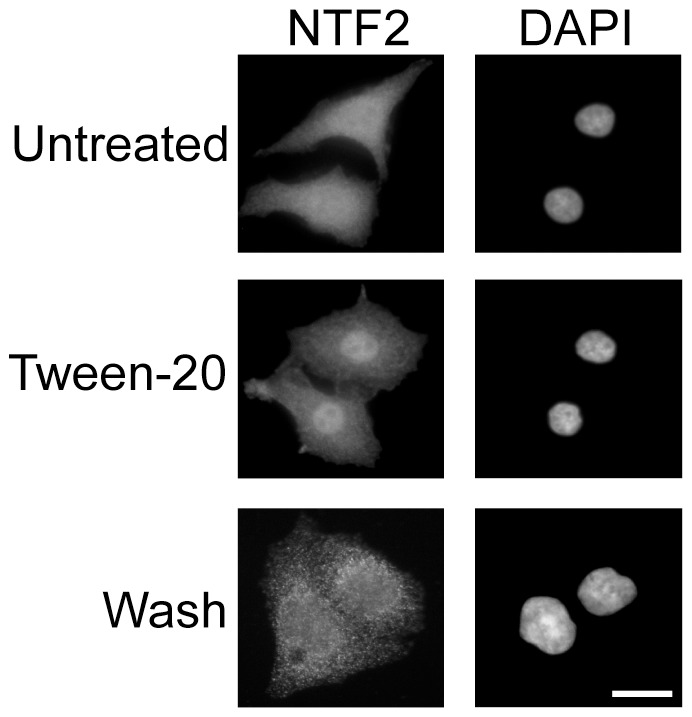
Tween-20 does not affect localization of RanGAP to the NPC. HeLa cells were incubated in serum-free DMEM (Untreated), serum-free DMEM containing 150 µM Tween-20 for 4 h (Tween-20), serum-free DMEM containing 150 µM Tween-20 for 4 h, washed and then placed in fresh serum-free DMEM for 1 h (Wash), or in serum-free DMEM containing 150 µM Tween-20 for 4 h and supplemented with 10% FBS (Serum add) and allowed to incubate for 1 h at 37°C. The cells were then washed and fixed in 1× PBS containing 4% paraformaldehyde before the distribution of RanGAP (left column) and FG repeat containing nucleoporins (mAb414, middle column) were monitored by immunofluorescence microscopy. Overlay analysis was performed to monitor any change in the localization of RanGAP (right column). Scale bar represents 10 µm.

Ran is transported from the cytoplasm to nucleus in the GDP form. Conversion of Ran in the GTP state to the GDP form in the cytoplasm requires activation of the GTPase activity of Ran by RanGAP. In mammalian cells RanGAP is found at the NPC at steady-state [14], [15]. Thus, it is possible that Tween-20 treatment affects localization of RanGAP to the NPC, causing Ran to remain in the GTP bound form and accumulate in the cytoplasm. Thus, immunofluorescence microscopy was used to monitor the cellular location of RanGAP in HeLa cells treated with 150 µM Tween-20 for 4 h in serum-free DMEM. The NPC was visualized by staining for nucleoporins using mAB414, a monoclonal antibody raised against a subset of FG-repeat containing Nups (Figure 8). RanGAP displays a typical nuclear rim staining in HeLa cells incubated in serum-free DMEM for 4 h (Figure 8). Moreover, the location of RanGAP did not change in cells treated with Tween-20. RanGAP also remained at the nuclear periphery when Tween-20 was removed from the cells by replacing Tween-20 containing serum-free DMEM with fresh serum-free DMEM, or when serum was added to Tween-20-containing DMEM. Co-localization analyses verify that RanGAP is located at the NPC in treated and untreated cells, suggesting that cytoplasmic accumulation of Ran is not caused by mislocalization of RanGAP.

**Figure 9:**
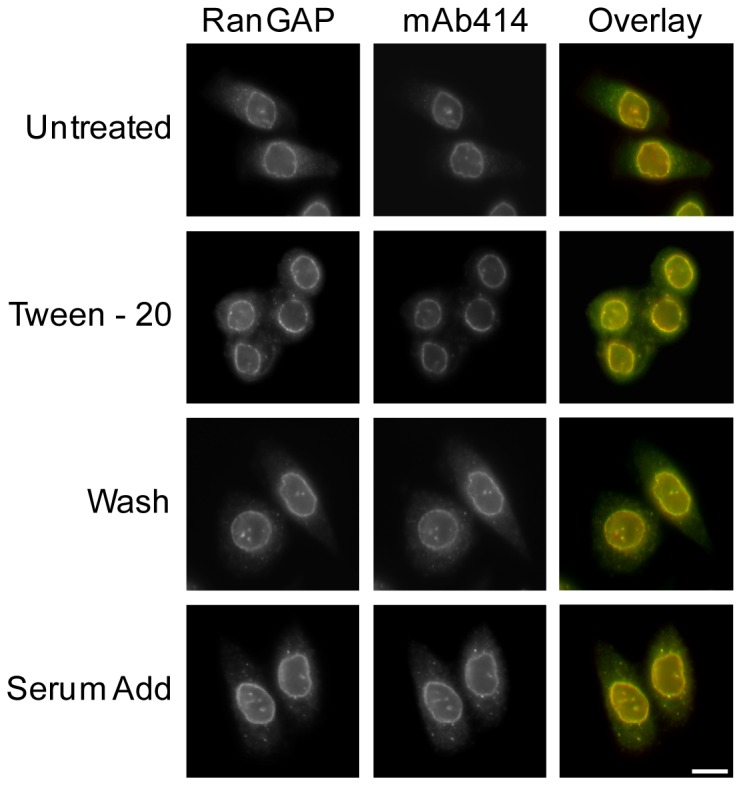
Tween-20 does not inhibit the enzymatic activity of Ran or RanGAP. Ran was incubated alone (•), in the presence of RanGAP (▾), in the presence of RanGAP that was treated with 150 µM Tween-20 for 30 min on ice prior to the start of the experiment (⧫), or was treated with 150 µM Tween-20 for 30 min on ice before the addition of RanGAP (▪). The rate of GTP hydrolysis by Ran was measured by the release of ^32^P by the charcoal method.

To investigate the possibility that Tween-20 affects the activities of Ran and/or RanGAP activation of the GTPase activity of Ran, the activity of each protein was tested *in vitro* using a GTP hydrolysis assay [9], [43], [44]. Recombinant Ran loaded with [γ-^32^P]GTP was incubated with or without 150 µM Tween-20 for 30 min prior to the addition of recombinant RanGAP. When RanGTP is incubated in the absence of RanGAP, very little GTP hydrolysis occurs, confirming that Ran has very low intrinsic GTPase activity ([12] and Figure 9). In contrast, when RanGTP is incubated with RanGAP the GTPase activity of Ran is stimulated and significant hydrolysis of GTP occurs. When RanGTP pre-incubated with Tween-20 was incubated with RanGAP, the rate of GTP hydrolysis was faster compared with that of the untreated enzyme. Similarly, when RanGTP was incubated with RanGAP pre-incubated with 150 µM Tween-20, the rate of hydrolysis of GTP observed was comparable to that of RanGAP that was not pre-incubated with Tween-20. These results show that Tween-20 does not inhibit the activities of Ran and RanGAP. The data suggest that accumulation of Ran in the cytoplasm is not due to a defect in the enzymatic activity of Ran or the function of RanGAP, and that Ran retained in the cytoplasm of Tween-20 treated cells is most likely in the GDP-bound form. However, why the rate of GTP hydrolysis increased when RanGTP or RanGAP was pre-incubated with Tween-20 is not understood.

### Tween-20 treatment of HeLa cells causes nuclear Accumulation of NTF2

**Figure 10:**
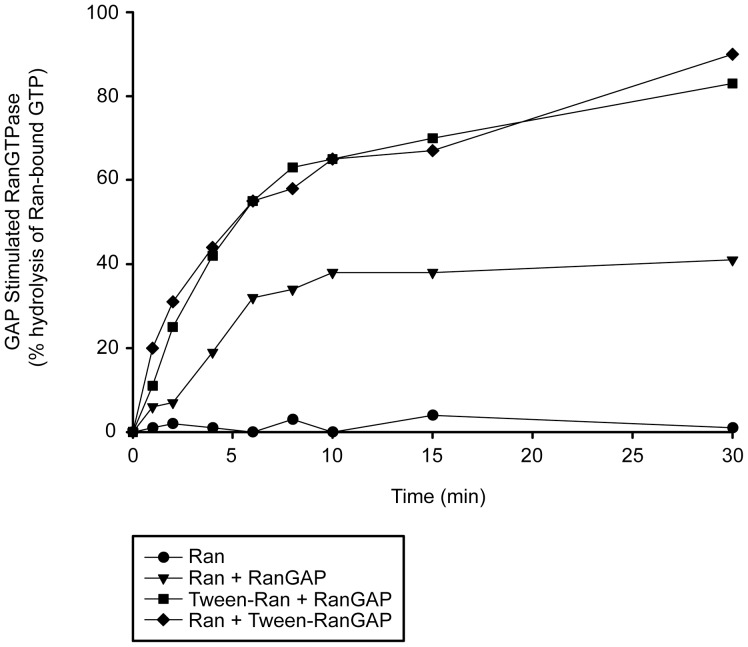
Tween-20 causes nuclear accumulation of NTF2. HeLa cells were incubated in serum-free DMEM without (Untreated) or with 150 µM Tween-20 for 4 h (Tween-20), or in serum-free DMEM with 150 µM Tween-20 for 4 h and then washed and incubated in serum-free DMEM without Tween-20 (washed) for 1 h. The cells were washed and fixed in 1× PBS containing 4% paraformaldehyde, and the distribution of NTF2 was monitored by immunofluorescence microscopy. The cells were stained with DAPI to visualize the nucleus. Scale bar represents 10 µm.

The importance of the Ran cycle for proper nuclear-cytoplasmic exchange of macromolecules has been well documented from yeast to mammal [39], [45], [46]. Upon hydrolysis of its bound GTP, RanGDP returns to the nucleus and is converted to RanGTP to facilitate another round of transport. The nuclear transport factor NTF2, is responsible for returning RanGDP to the nucleus [19], [20]. We therefore investigated whether mislocalization of Ran to the cytoplasm during Tween-20 treatment was the result of an altered NTF2 localization. Immunofluorescence microscopy analysis shows that NTF2 localizes to both the nucleus and cytoplasm in untreated HeLa cells ([18], [26] and Figure 10). However, in cells treated with 150 µM Tween-20 for 4 h, the distribution of NTF2 changes and becomes predominantly nuclear. In Tween-20 treated cells incubated in serum-free DMEM lacking Tween-20, NTF2 re-distributes between the nucleus and cytoplasm, but the NTF2 signal in these cells is somewhat different from that obtained in untreated cells. The reason for this change is not known. Nevertheless, the data show that Tween-20 treatment causes nuclear accumulation of NTF2, and suggest that retention of NTF2 in the nucleus may cause Ran to remain in the cytoplasm of cells treated with Tween-20.

### Overexpression of NTF2 restores nuclear import of Ran and nuclear export of tRNA and NES-EGFP in Tween-20 treated HeLa cells

**Figure 11:**
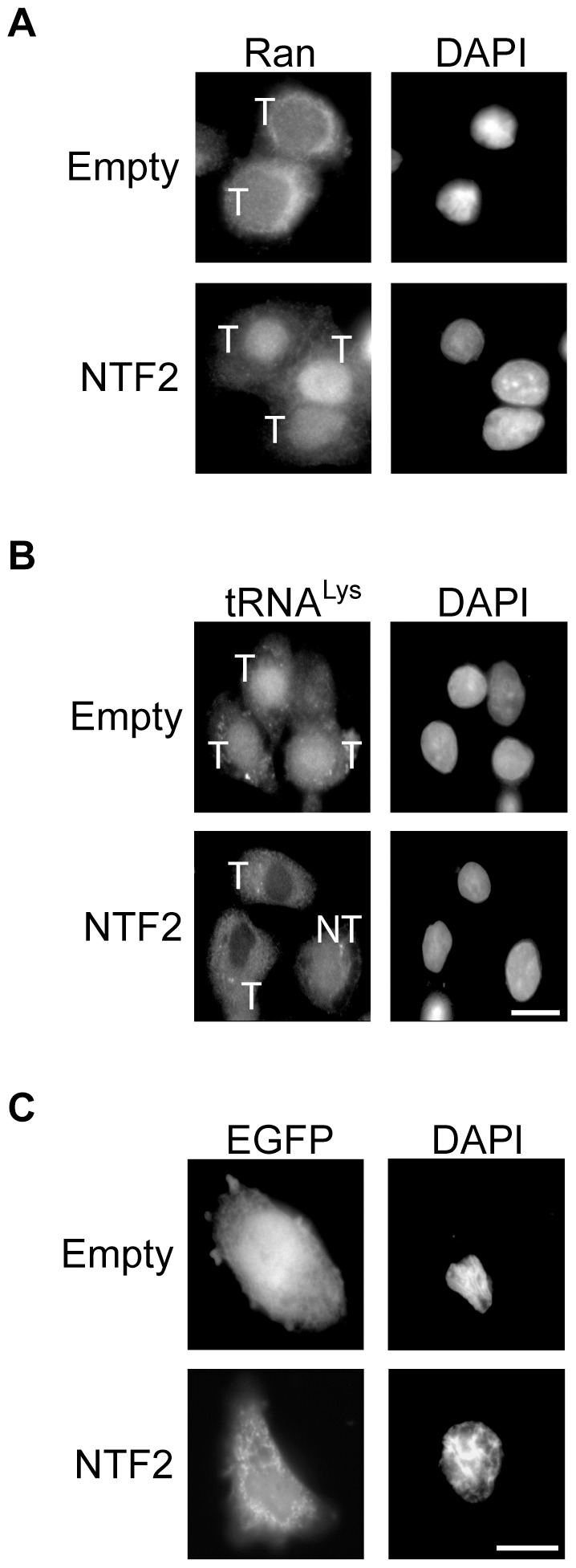
Overexpression of NTF2 restores nuclear import of Ran and nuclear export processes in Tween-20 treated HeLa cells. HeLa cells were transfected with pCMV (Empty) or pCMV-NTF2 (NTF2) for 24 h. The cells were washed and incubated in fresh serum-free DMEM containing 150 µM Tween-20 for 4 h. The cells were then washed and fixed in 1× PBS containing 4% formaldehyde. (A) The cellular location of Ran was monitored by immunofluorescence microscopy or (B) the location of tRNA^Lys^ was detected by FISH. (C) HeLa cells were co-transfected with NES-EGFP and pCMV or pCMV-NTF2. 24 h post-transfection, the cells were incubated in serum-free DMEM with 150 µM Tween-20 for 4 h and the cellular location of NES-EGFP was monitored by fluorescence microscopy. The nuclei were visualized by DAPI staining. T, transfected cell; NT, non-transfected cell. Scale bars represent 10 µm.

To verify that Tween-20 is affecting a mechanism that regulates nuclear export of NTF2, the effect of overexpression of NTF2 on nuclear import of Ran in Tween-20 treated HeLa cells was investigated (Figure 11A). HeLa cells were transfected with the pCMV vector alone or pCMV containing the gene for NTF2 and incubated in serum-free DMEM containing 150 µM Tween-20 for 4 h (Figure 11A). Immunofluorescence microscopy shows that cells transfected with the pCMV vector alone (T) and treated with Tween-20 retained Ran in the cytoplasm (panel A, Empty). In contrast, Ran was found primarily in the nucleus in pCMV-NTF2 transfected cells (T) exposed to Tween-20 (panel A, NTF2). The data suggest that cytoplasmic accumulation of Ran is caused by Tween-20 inhibiting a mechanism that facilitates translocation of NTF2 to the cytoplasm.

To substantiate that restoration of nuclear import of Ran by overexpression of NTF2 restores the nuclear export processes, nuclear export of tRNA and NES-EGFP was monitored in Tween-20 treated HeLa cells overproducing NTF2. The analyses show that both tRNA^Lys^ (panel B) and NES-EGFP (panel C) are retained in the nucleus of Tween-20 cells that were transfected with the empty plasmid (T). In contrast, Tween-20 treatment did not affect nuclear export of tRNA^Lys^ and NES-EGFP in cells overproducing NTF2 (T). In cells that were not transfected with the pCMV-NTF2 plasmid (NT), tRNA^Lys^ was retained in the nucleus (Figure 11B). These findings are also consistent with overexpression of NTF2 restoring nuclear import of Ran in Tween-20 treated cells. Furthermore, collectively the data suggest that the primary effect of Tween-20 is on translocation of NTF2 from the nucleus to the cytoplasm.

### Tween-20 treatment results in increased phosphorylation of phospho-tyrosine and phospho-threonine proteins, but does not appear to cause modification to NTF2

**Figure 12:**
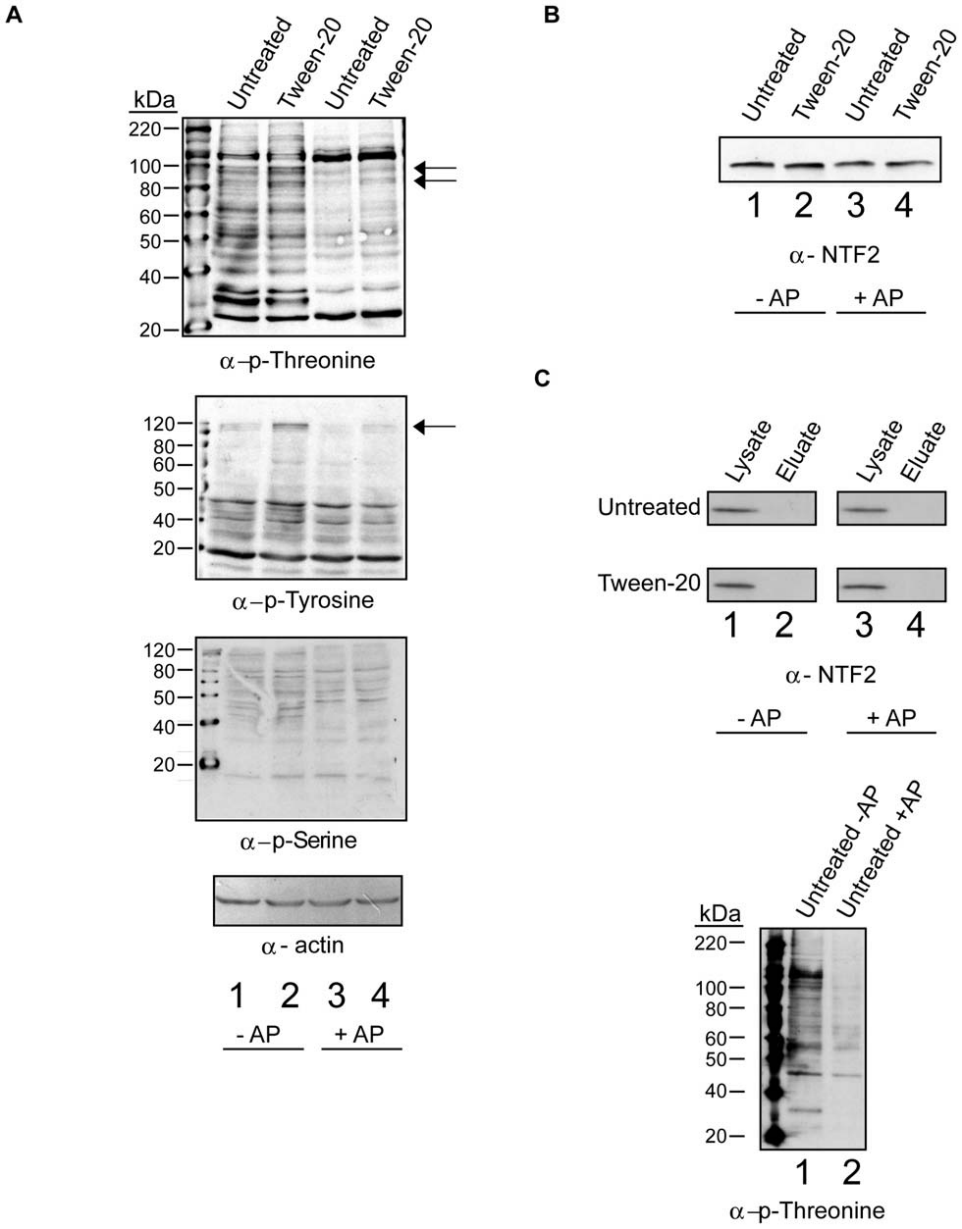
Tween-20 causes an increase in the level of protein phosphorylation at tyrosine and threonine residues, but does not appear to cause modification of NTF2. (A) HeLa cells were incubated in serum-free DMEM without (Untreated) or with 150 µM Tween-20 (Tween-20) for 4 h. The cells were washed and lysed in the presence of sodium fluoride and sodium orthovanadate (Lanes 1 and 2, -AP) or lysed in the absence of phosphatase inhibitors (Lanes 3 and 4, +AP). The cells lysed without phosphatase inhibitors were incubated with alkaline phosphatase for 30 min at 37°C. Following the incubation, 40 µg of each lysate was separated on 10% gels by SDS-PAGE followed by Western blot analysis to monitor the levels of protein phosphorylation at threonine (first row), tyrosine (second row) and serine (third row) residues or actin level (fourth row). Arrows indicate bands of increased phosphorylation. (B) The lysate (40 µg) prepared as in (A) was separated by SDS-PAGE on a 15% gel followed by Western blot analysis to monitor the mobility of NTF2. The relative mobility of NTF2 was monitored in lysate prepared from Untreated (lanes 1 and 3) and Tween-20 (lanes 2 and 4) treated cells. (C) Lysates treated as in (A) were subjected to chromatography using Phostag™-agarose. The resins were washed and bound proteins were eluted using sodium phosphate containing buffer. Total cell lysate (20 µg) (lanes 1and 3, left) and eluted proteins were subjected to Western blot analysis. NTF2 was detected using α-NTF2 (left) and phospho-Thr proteins (lanes 1 and 2, right) were detected with anti-phospho-Thr antibodies.

A number of signaling mechanisms have been implicated in controlling the localization of Ran and various β-karyopherins in mammals [26], [27], [39]. To investigate the possibility that a phosphorylation-dependent signal is involved in nuclear retention of NTF2 upon Tween-20 treatment, Western blot analysis was performed on cell lysates prepared from untreated or Tween-20 treated HeLa cells using anti-phospho-threonine, anti-phopho-serine and anti-phospho-tyrosine antibodies (Figure 12A). Several phospho-threonine (first row, lane 1), phospho-tyrosine (second row, lane 1), and phospo-serine (third row, lane 1) proteins were detected in cell lysate prepared from untreated cells. The phosphorylation level of at least two of the phospho-threonine proteins (first row, lane 2) and one phospho-tyrosine protein (second row, lane 2) were increased by 4- and 10-fold respectively, in cell lysate prepared from Tween-20 treated cells; however no change was detected in the phospho-serine protein levels (third row, compare lanes 1 and 2). Analysis of the actin levels by Western blot indicated the same amount of cell extract was used (fourth row, lanes 1–4). Verification that these proteins are phosphorylated was obtained by Western blot analysis of the cell lysates treated with alkaline phosphatase (lanes 3 and 4, top and bottom). The increased phosphorylation signal of the proteins that appear in the Tween-20 treated lysate without alkaline phosphatase decreased significantly upon treatment with alkaline phosphatase (compare lane 2 and lane 4, top and bottom row). This indicates that the increase in phosphorylation of the phospho-threonine and phospho-tyrosine proteins was the result of phosphorylation events triggered by Tween-20 treatment of the cells. Moreover, based on size none of these proteins are Ran and NTF2. Furthermore, the involvement of a serine kinase in the response to Tween-20 can be ruled out, as this treatment did not affect phosphorylation of phospho-serine proteins (third row, lane 2).

To investigate the possibility that NTF2 is modified during Tween-20 treatment (Figure 12B), Western blot analysis was used to monitor the mobility of NTF2 in cell lysates prepared from untreated and Tween-20-treated HeLa cells. The mobility of NTF2 (compare lanes 1 and 2) on a 15% SDS-PAGE gel did not change in response to Tween-20 treatment. Furthermore, the mobility of NTF2 did not change in alkaline phosphatase treated cell lysates prepared from untreated and Tween-20 treated cells (lanes 3 and 4). In addition the level of NTF2 was not affected by Tween-20 treatment (compare lanes 1 and 2). These findings suggest that NTF2 may not be phosphorylated.

To verify by an alternative strategy that NTF2 is not phosphorylated in untreated and Tween-20 treated cells, phosphorylated proteins were first isolated by chromatography using Phostag™-agarose resin followed by Western blot analysis of the isolated proteins to detect NTF2 (Figure 12C, left panel). Phostag™ binds to phosphates attached to various macromolecules including Ser, Thr and Tyr residues of proteins. For this analysis, total cell lysate was prepared from untreated and Tween-20 treated HeLa cells in the presence or absence of phosphatase inhibitor. The lysates prepared in the absence of inhibitor were incubated with alkaline phosphatase to cleave the phosphate groups linked to proteins. The lysates were then incubated with Phostag™- agarose and after the resin was washed to remove unbound proteins, the bound proteins were eluted with sodium phosphate containing buffer. NTF2 was detected in total cell lysate (lanes 1 and 3) prepared from untreated and Tween-20 treated cells. However, NTF2 was not detected in the eluates (lane 2) from the Phostag™-agarose resin incubated with cell lysate prepared from untreated and Tween-20 treated cells, or in eluates obtained from Phostag™-agarose incubated with alkaline phosphatase treated cell lysates (lane 4). A large number of phospho-Thr proteins were found in eluates from the resin incubated with total cell lysate prepared from untreated cells (right panel, lane 1). The level of these proteins in eluates from the resins incubated with cell lysate treated with alkaline phosphatase (lanes 2) decreased considerably, indicating that the Phostag™-agarose strategy is capable of isolating phosphorylated proteins. Thus, these results suggest that NTF2 may not be phosphorylated in response to Tween-20 treatment. Moreover, these data suggest that Tween-20 may be influencing a tyrosine and/or threonine kinase signal transduction pathway that could be involved in regulating translocation of NTF2 to the cytoplasm.

### The ERK and PI3K signaling pathways do not appear to control nuclear-cytoplasmic trafficking of NTF2

**Figure 13:**
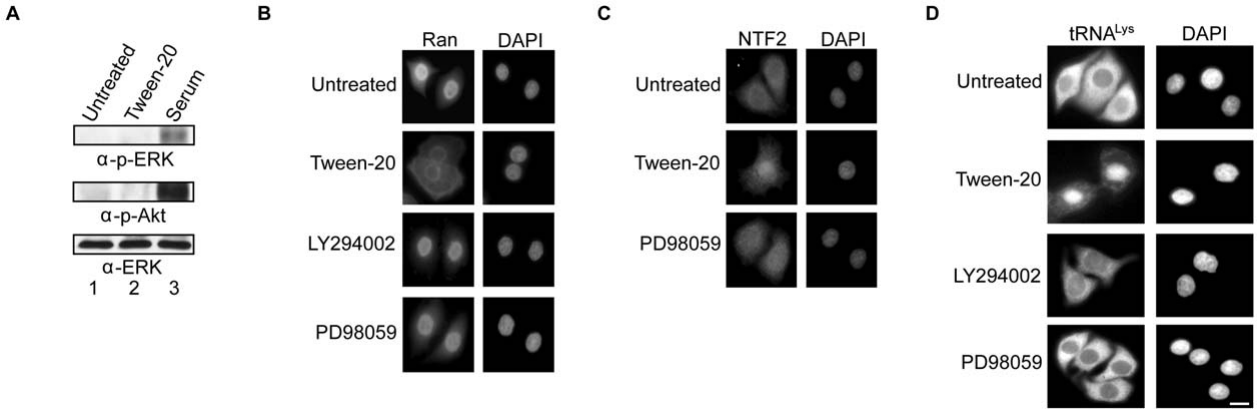
The ERK and PI3K signaling pathways do not control nuclear-cytoplasmic trafficking of NTF2. (A) HeLa cells were incubated in serum-free DMEM for 4 h (lane 1) and then maintained in serum-free DMEM alone for 4 h, serum-free DMEM containing 150 µM Tween-20 for 4 h (lane 2), or in serum-free DMEM for 4 h and then stimulated with 10% FBS for 30 min (serum) (lane 3). The cells were washed and lysed in the presence of phosphatase inhibitors. 40 µg of each lysate was separated by SDS-PAGE followed by Western blot analysis to monitor the levels of phosphorylated ERK1/2 (p-ERK1/2) (top row) or phosphorylated Akt (p-Akt) (middle row). Analysis of the levels of ERK1/2 (bottom row) was performed as an internal loading control. (B, C and D) HeLa cells were incubated in serum-free DMEM (Untreated), serum-free DMEM containing: 150 µM Tween-20 (Tween-20), 25 µM LY294002 or 50 µM PD98059 for 4 h. The cells were washed and processed for immunofluorescence microscopy to monitor the localization of (B) Ran and (C) NTF2, or for FISH to monitor the localization of tRNA^Lys^ (D). The nuclei were visualized by DAPI staining. Scale bar represents 10 µm.

The ERK and PI3K pathways have previously been implicated in controlling the location of various transport components in response to certain stress [27], [28], [34]. As reported previously, these pathways are turned off when HeLa cells are starved of serum (Figure 13A, lane 1) and are turned on by exposing the serum-starved cells to serum (lane 3) based on phosphorylation of ERK (top panel) and Akt (middle panel). Furthermore, Tween-20 treatment of the serum-starved cells in serum-free DMEM had no effect on the phosphorylation status of ERK and Akt (lane 2), indicating that Tween-20 does not activate the ERK or PI3K pathways. We have also established that the localization of NTF2, Ran, tRNA and reporter proteins is not affected when HeLa cells are incubated in serum-free DMEM for 4 h or over an 8 h period (data not shown), confirming that turning off these pathways has no effect on these processes. These findings suggest that the ERK and PI3K pathways may not play a role in regulating translocation of NTF2 to the cytoplasm. Nevertheless, to verify by an alternative strategy that these pathways are not involved in regulating translocation of NTF2 to cytoplasm, nuclear-cytoplasmic trafficking of Ran (panel B), NTF2 (panel C) and tRNA (panel D) was monitored in HeLa cells incubated in serum-free DMEM or serum-free DMEM containing 150 µM Tween-20, 50 µM LY294002 or 25 µM PD98059. In cells incubated in serum-free DMEM nuclear-cytoplasmic trafficking of Ran, NTF2 and tRNA was not affected (panels B, C and D). Consistent with previous experiments, Tween-20 treatment affects trafficking of Ran, NTF2 and tRNA (panels B, C and D). Treatment of the cells with the PI3K pathway inhibitor, LY294002, or with the MEK1/2 inhibitor of the ERK pathway, PD98059, did not affect the localization of Ran (panel B). Furthermore, when cells were treated with PD98059 or LY294002 (data not shown) NTF2 displayed an even cellular distribution similar to its location in untreated cells (Figure 13C, compare untreated and PD98059). Similarly, no effect was observed on the location of tRNA^Lys^ when HeLa cells were treated with PD98059, LY294002 (panel D), or the p38 MAPK inhibitor SB202190 (data not shown). Taken together, the data suggest that Tween-20 may be affecting an ERK1/2- and PI3K-independent signaling pathway that controls regulation of nuclear-cytoplasmic translocation of NTF2.

### Prolonged Tween-20 treatment leads to apoptosis

**Figure 14:**
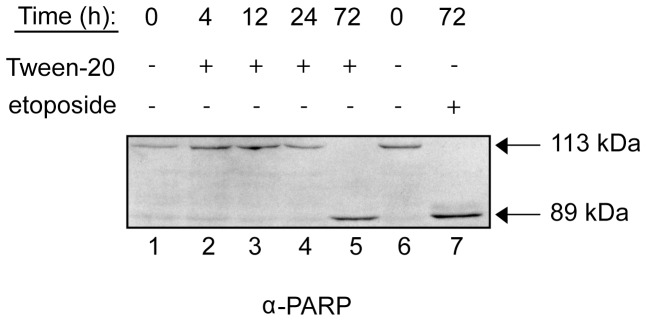
Prolonged Tween-20 treatment leads to apoptosis. HeLa cells were incubated in serum-free DMEM (Untreated), serum-free DMEM containing 150 µM Tween-20, or serum-free DMEM containing 25 µM etoposide. The cells were harvested at the time points indicated, lysed and 40 µg of cell lysate was separated by electrophoresis on a 10% SDS-PAGE gel. Western blot analysis was performed to monitor PARP cleavage.

To directly test whether HeLa cells treated with Tween-20 undergo apoptosis, we monitored the cleavage of poly ADP-ribose polymerase (PARP). PARP is a nuclear enzyme that responds to DNA damage and facilitates repair [47], [48]. PARP can also be used as a marker for apoptosis, since activation of apoptosis leads to cleavage of full-length PARP (113 kDa) by Caspase-3 and -7 into two smaller fragments (89 and 24 kDa); the 113 kDa and 89 kDa forms are readily distinguished by Western blot analysis [49], [50]. To test whether Tween-20 treatment triggers an apoptotic response, cells were seeded on 10 cm dishes and grown overnight. The cells were washed and incubated in fresh serum-free DMEM with or without Tween-20. Cells were lysed at 0, 4, 12, 24, and 72 h post-treatment and the cleavage of PARP was monitored by Western blot analysis (Figure 14). Full-length PARP was detected in untreated cells and in cells treated with Tween-20 for up to 24 h (lanes 1–4). However, PARP cleavage was detected in lysate prepared from cells treated with Tween-20 for 72 h, indicating that programmed cell death had been initiated (lane 5). This initiation of apoptosis was also observed when the cells were treated with the DNA damaging agent etoposide (25 µM) for 72 h (lane 7), as cleavage of PARP was detected in lysate from etoposide treated cells. The data indicate that Tween-20 triggers the cleavage of PARP and activation of an apoptotic response although the time required to trigger this response is long after the observed effect on nuclear export of tRNA, mRNA, and protein, and nuclear import of Ran, suggesting that inhibition of these processes, in part, leads to apoptosis.

## Discussion

The main signaling pathway in eukaryotes that senses changes in nitrogen availability is TORC1. TORC1 is active when nitrogen sources are available and turned off under conditions of low nitrogen. Recent studies have shown that the TORC1 signaling pathway of *S. cerevisiae* is, in part, regulating nuclear export of mature spliced tRNAs in response to availability of nitrogen sources, as inhibition of TORC1 with rapamycin resulted in inhibition of nuclear export of spliced tRNAs [30], [31]. In contrast to *S. cerevisiae*, rapamycin treatment did not affect nuclear tRNA export in mammalian cells, suggesting that TORC1 signaling pathway may not regulate nuclear tRNA export in mammals. This is consistent with the finding that amino acid deprivation also does not affect nuclear-cytoplasmic trafficking of tRNAs in mammalian cells [29]. However, a surprising finding from this investigation is that Tween-20, a component of the solution used to deliver rapamycin to mammalian cells, affects nuclear-cytoplasmic trafficking processes.

Tween-20 inhibits nuclear export of tRNA, Importin-α, which is the cargo for the nuclear export β-karyopherin CAS, and a NES-EGFP reporter, which is known to be a Crm1 substrate. However, Tween-20 treatment did not affect nuclear import of the nuclear tRNA export receptor Xpo-t, Importin-α, which participates in nuclear import of proteins with classical NLSs, NES-EGFP, the NLS-containing Histone H2A or NF-κB. These data suggest that Tween-20 only affects nuclear export processes. Tween-20 treatment also caused accumulation of Ran in the cytoplasm and NTF2 in the nucleus, the receptor responsible for nuclear import of Ran in the GDP-bound form. Moreover, the nuclear export processes and nuclear import of Ran were restored when the Tween-20 stimulus was removed. These effects were only observed with Tween-20, as treatment of cells with other detergents such as deoxycholate, Triton X-100 (data not shown) and Tween-80 did not affect the nuclear export processes. The unavailability of Ran in the nucleus is most likely the reason for inhibition of nuclear export of tRNA and proteins during Tween-20 treatment. The absence of Ran in the nucleus would result in an inability of the nuclear export receptors Xpo-t, CAS and Crm1 to bind their cargoes, a process that is known to be facilitated by RanGTP. Accumulation of Ran in the cytoplasm is unlikely to be caused by Tween-20 affecting activation of the GTPase activity of Ran in the cytoplasm based on the following observations: (a) localization of RanGAP to the NPC was not affected by Tween-20 treatment, and (b) Tween-20 did not affect the function of RanGAP in activation of the GTPase activity of Ran, or the Ran GTPase activity *in vitro*. The most likely explanation is that nuclear accumulation of NTF2 is the cause of the buildup of Ran in the cytoplasm during Tween-20 treatment. This interpretation is consistent with the finding that overexpression of NTF2 restored nuclear import of Ran and nuclear export of tRNA and the NES-EGFP chimera in Tween-20 treated cells, and with previous reports showing that microinjection of antibodies against the C-terminus of NTF2 in mammalian cells results in cytoplasmic accumulation of Ran [51]. The mechanism by which Tween-20 causes NTF2 to accumulate in the nucleus is not understood. However, this is unlikely to be due to a clogging of the nuclear pore complex, since nuclear import of NLS-containing substrates is unaffected, and overexpression of NTF2 was able to alleviate the export defects imposed by Tween-20 treatment. Nevertheless, this property of Tween-20 facilitated the discovery of a previously unrealized mechanism that regulates nuclear-cytoplasmic translocation of NTF2.

The entire Tween-20 molecule is required to elicit nuclear accumulation of NTF2. Treatment of HeLa cells with either the lauric acid, or polyethylene glycol component of Tween-20 was unable to affect the nuclear export processes, as nuclear tRNA export was not affected. Similarly, sorbitan monolaurate did not affect nuclear tRNA export. These findings suggest that the overall amphipathic property of Tween-20 may play a role in influencing the action of Tween-20, or its location and activity. While the location and action of Tween-20 are not known, it is possible that this occurs at the plasma membrane level by insertion into the membrane where it interacts non-covalently with a membrane-bound receptor of a signaling cascade, which transmits the signal to the nucleus, leading to inhibition of nuclear export of NTF2. Alternatively, monomeric Tween-20 in solution outside of the cells could be interacting with the ligand binding site of a membrane-bound receptor to affect signaling. These possibilities are consistent with the finding that removal of Tween-20 by washing treated cells restores proper localization of Ran and NTF2 as well as nuclear export of tRNA and mRNA. Furthermore, the reported effect of Tween-20 on the Ran-dependent nuclear export processes is not due to a disruption of membrane integrity as Tween-20 treated cells were just as capable as untreated cells to exclude trypan blue (data not shown).

Tween-20 but not Tween-80 or Tween-21 (data not shown) caused nuclear accumulation of NTF2, suggesting that the effects observed are specific for Tween-20. However, a more likely explanation for the inability of Tween-80 and Tween-21 to affect translocation of NTF2 to the cytoplasm is related to their CMCs. The CMCs of Tween-20, Tween-80 and Tween-21 are 80, 12 and 5 µM, respectively; thus, it is possible that the minimal monomeric concentration of a Tween detergent that is required to inhibit nuclear export of NTF2 is 80 µM and this may be the reason why an effect was not observed by the Tween detergents with CMCs lower than this concentration, as they would exist primarily in the micellar form at that concentration. This interpretation is in agreement with the finding that Tween-20 has no effect on nuclear-cytoplasmic trafficking of NTF2 when the analysis was performed with 5 or 12 µM Tween-20 (data not shown). Thus, it is likely that Tween-20 is not the only member of the Tween family of detergents that can affect nuclear export of NTF2. Nevertheless, it is clear that inhibition of nuclear export of NTF2 by Tween-20 is specific, as detergents outside of the Tween family with higher CMC did not affect nuclear export processes.

Tween-20 treatment of HeLa cells resulted in increased phosphorylation of both phospho-tyrosine and phospho-threonine proteins, indicating that Tween-20 is affecting a signal transduction pathway(s) that may be responsible for controlling regulation of nuclear export of NTF2. This pathway does not appear to be p38 MAPK (data not shown), PI3K/Akt or MEK1/2 of the ERK pathway, as inhibition of these pathways had no effect on nuclear export of tRNA and NTF2, or nuclear import of Ran. It is possible that Tween-20 is affecting more than one pathway or a pathway involving both threonine and tyrosine kinases. However, it is unclear whether Tween-20 leads to activation or inhibition of the putative signal transduction pathway(s) to cause a block in nuclear export of NTF2. Furthermore, it is not known how this pathway regulates transport of NTF2 from the nucleus to the cytoplasm, and how Tween-20 affects this regulatory mechanism. It is unlikely that NTF2 is directly controlled by phosphorylation, as phosphorylated NTF2 was not detected in cell lysate obtained from untreated and Tween-20 treated HeLa cells, or in isolates of phosphorylated proteins purified by chromatography using Phostag™-agarose resin. Regulation of translocation of NTF2 to the cytoplasm could be achieved by two possible mechanisms. The first is that the function of a nuclear protein, which is regulated, is responsible for controlling the nuclear-cytoplasmic trafficking activity of NTF2, and Tween-20 treatment may lead to inhibition or activation of the function of this protein, causing inhibition of NTF2 translocation to the cytoplasm. The second is that interaction between NTF2 and specific nucleoporins on the nucleoplasmic side of the NPC is regulated by reversible modification of the nucleoporins, as the transport activities of receptors have been shown to be affected by phosphorylation of Nups [52]–[54]. However, this modification is unlikely to result in the degradation of the specific Nups as mAb414 staining was unaltered in cells and *in vitro* by Western blot analysis (data not shown). Tween-20 treatment may lead to the disruption of the hydrophobic core of the NPC resulting in a loss of the permeability barrier. However, this is also unlikely to be the case as nuclear import of NLS containing proteins was not affected during Tween-20 treatment. Determination of the identities of the phospho-threonine and phospho-tyrosine proteins will facilitate the identification of the signal transduction pathway controlling regulation of nuclear-cytoplasmic trafficking of NTF2 to the cytoplasm, and thus leading to an understanding of how Tween-20 may affect this pathway.

mRNA export proceeds mainly by a RanGTP-independent pathway that involves the export proteins TAP/Mex67p and Nxt1/Mtr2p [55]. However, RanGTP does play a role in Crm1-mediated export of a subset of mRNAs [55]. Previously it was shown in *S. cerevisiae* that expression of a GTPase-deficient mutant form of Ran resulted in a nuclear accumulation of mRNA due to the inability of the cells to hydrolyze GTP [56]. Furthermore, expression of a temperature-sensitive RCC1 allele in the tsBN2 cell line also resulted in nuclear accumulation of mRNA in mammals [57]. In both cases the defect observed in mRNA transport appears to be the result of an altered RanGTP cycle. Although mRNA export is not directly dependent on RanGTP, Tween-20 treatment resulted in inhibition of nuclear mRNA export. Redistribution of mRNA to the cytoplasm was observed when Tween-20 was removed from the media. Thus, it is not known how Tween-20 caused a block in nuclear mRNA export. One possibility is that Tween-20 leads to inhibition of a step required for nuclear export of mRNA. Alternatively, release of a protein involved in mRNA splicing and/or export from the nuclear import receptor is blocked because of the unavailability of RanGTP in the nucleus, as it is known that release of the cargo from a nuclear import β-karyopherin requires RanGTP binding to the receptor [16].

NTF2 has been shown to accumulate in the nucleus of keratinocytes as the cells began to undergo apoptosis during etoposide treatment [58]. NTF2 accumulation in the nucleus during Tween-20 treatment was not due to a commencement of the programmed cell death pathway, as cleavage of PARP was not observed in HeLa cells exposed to Tween-20 for 4 h. The finding that HeLa cells treated with Tween-20 for 4 h retained the ability to redistribute the mislocalized proteins to their proper compartment within minutes of removal of the Tween-20 stimulus, also suggests that the cells had not begun to undergo apoptosis. However, HeLa cells undergo apoptosis after exposure to Tween-20 for 72 h based on cleavage of PARP, which is the same time required for etoposide to induce apoptosis. The effects of Tween-20 treatment also differ from the effects of etoposide treatment reported by Suh *et al.* (2004), as they also detected nuclear accumulation of Ran. The fact that cytoplasmic retention of Ran occurred during Tween-20 treatment suggests that the mode of action of Tween-20 and etoposide on these cell lines may be different.

The exchange of macromolecules between the nucleus and cytoplasm is absolutely required for the survival of the cell. Leptomycin B and derivatives are the only known inhibitors of the nuclear-cytoplasmic trafficking process in yeast and mammals. Leptomycin B binds covalently to Crm1 and prevents an interaction with its NES containing substrate. We demonstrate here the first case of an inhibitor that blocks all Ran-mediated nuclear export processes. Tween-20 most likely acts at the membrane level resulting in the transmission of a signal that causes a reversible block in the export of NTF2 to the cytoplasm. Nuclear retention of NTF2 causes Ran to accumulate in the cytoplasm leading to the observed defect in all Ran-mediated nuclear export processes. Finally, this study demonstrates for the first time that nuclear export of NTF2 is regulated and that Tween-20 can be used as a tool to identify and characterize this mechanism.

## Materials and Methods

### Cell culture and transfections

HeLa and rat hepatoma H4IIE cells were purchased from the ATCC. Cells were maintained in DMEM containing 10% fetal bovine serum at 37°C in the presence of 5% CO_2_ unless stated otherwise. Transfections were carried out using Lipofectamine 2000 (Invitrogen, Carslbad,CA) as per the manufacturer's instructions. Tween-20, sorbitan monolaurate (SPAN20), lauric acid, Tween-80, deoxycholate, rapamycin, PD98059, LY294002, SB202190, polyethylene glycol and etoposide were all purchased from Sigma-Aldrich (St. Louis, MS).

### Plasmids and antibodies

pQE32-RAN and pET23a-RANGAP were described previously [59]. The NES-EGFP was a generous gift from Dr. R. Mahieux (Pasteur Institute, Paris, France) [40]. pET23b-NTF2 was a generous gift from Dr. B. Paschal (University of Virginia, Charlottesville, USA) [18]. pCMV-NTF2 was generated by PCR amplification of the NTF2 ORF from pET23b-NTF2 and insertion into the PstI and SalI sites of pCMVTag2A (Stratagene). Anti-Ran was purchased from BD Transduction laboratories (Mississauga, Canada). RanGAP and Importin-α antibodies were purchased from Santa Cruz (Santa Cruz, CA), mAb414 was obtained from Covance (Princeton, NJ), ERK1/2, p-ERK1/2, p-Akt and NF-κB antibodies were from Cell Signaling (Danvers, MA), anti-NTF2 and anti-histone H2AX were obtained from AbCam (Cambridge, MA), anti-PARP was from Roche Applied Science (Indianapolis, IN), anti-phospho-serine, anti-phospho-threonine, and anti-phospho-tyrosine antibodies were purchased from Invitrogen. Anti-actin was purchased from Sigma-Aldrich. Rabbit polyclonal antibodies to Xpo-t were generated against recombinant Xpot-His_6_ and affinity purified (Invitrogen).

### Fluorescence in situ hybridization

HeLa or rat hepatoma H4IIE cells were fixed as described at http://www.singerlab.org/protocols and [59]–[61]. Fixed cells were rinsed with 1× PBS and permeabilized in 1× PBS containing 0.5% Triton X-100 (v/v) for 10 min on ice. The cells were rehydrated for 5 min in 4× SSC containing 50% formamide (v/v) and hybridized overnight at 37°C with 5 pmol of Cy3-labeled 5′-CGAACCCACGACCCTGAGATTAAGAGTCTCATGC-3′ to detect HeLa tRNA^Lys^_CUU_, Cy3-labeled 5′-CCAACGTGGGGCTCGAACCCACGACCCTGAGATTAAGAGTCTC ATGCTCTACCGACT-3′ to detect rat tRNA^Lys^_CUU_ or an AlexaFluor488-labeled oligo (dT) probe to detect mRNA in hybridization buffer consisting of 4× SSC, 50% formamide (v/v), 10% dextran sulfate (w/v), 0.2% BSA (w/v), 125 µg/ml *E. coli* tRNA, 500 µg/ml sheared salmon sperm DNA, and 2 mM vanadyl ribonucleoside complex. The cells were then washed at room temperature with 4× SSC containing 50% formamide, 2× SSC containing 50% formamide, 1× SSC containing 50% formamide, and 0.5× SSC containing 50% formamide. The cells were then stained with 1 µg/ml DAPI, washed 3 times with 1× PBS and mounted using Dako Cytomation (Roche). The human tRNA^Lys^ oligonucleotide used was designed using the tRNAscan-SE database [62] as described previously (5) and the rat tRNA^Lys^ oligonucleotide was described previously [33]. The cells were viewed with a NIKON Eclipse E600 fluorescent microscope with a 60× oil immersion objective. Images were captured using a CoolSnapFX CCD camera and analyzed using the MetaMorph Imaging Software.

### Immunofluorescence microscopy

Cells grown on coverslips were fixed with 4% paraformaldehyde in 1× PBS for 20 min at room temperature. The cells were permeabilized in 1× PBS containing 0.5% Triton X-100 (v/v) for 10 min on ice and then washed with 1× PBS. Cells were blocked in 1× PBS containing 5% skim milk for 1 h while shaking. The cells were then washed 3× with 1× PBS containing 0.1% Tween-20 (PBS-T). α-Ran (1∶50), α-RanGAP (1∶50), mAb414 (1∶50), α-NTF2 (1∶50), α-Importin-α (1∶50), α-NF-κB (1∶50), or α-Histone H2A (1∶50) was incubated with the cells in PBS-T for 1 h at 37°C. The cells were then washed 3× with PBS-T and subsequently incubated with the appropriate AlexaFluor conjugated secondary antibody (1∶2000) (Invitrogen) in PBS-T containing 5% skim milk for 1 h at room temperature. The cells were then washed 3× with PBS-T and stained with DAPI and mounted as described above.

### Expression and purification of His-tagged proteins

*E. coli* M15pREP4 transformed with pQE32-RAN or *E. coli* BL21 CodonPlus transformed with pET23a-RANGAP was grown at 37°C in 1 L of 2YT medium containing 100 µg/ml ampicillin and 20 µg/ml kanamycin or 34 µg/ml chloramphenicol was used instead of kanamycin in *E. coli* BL21 CodonPlus (5). The cells were grown to an OD_600_ of 0.3, and expression of the fusion proteins was induced with 500 µM isopropyl-β-D-thiogalactoside (IPTG) overnight at 15°C. The cells were harvested and resuspended in binding buffer (20 mM NaH_2_PO_4_, pH 7.5, containing 500 mM NaCl and 5 mM imidazole), and a mixture of protease inhibitors (Complete EDTA-free; Roche Applied Science). The cells were lysed with 2 passes through a French Press at 10,000 psi, and clarified lysates were loaded onto 1 ml HisTrap HP columns (GE Healthcare, Little Chalfont, Buckinghamshire, United Kingdom). RanGAP was expressed and purified as previously described [63]. For Ran, the column was washed with 40 ml of tRNA wash buffer (20 mM NaH_2_PO_4_, pH 7.5 containing 500 mM KCl and 5 mM imidazole) to remove any tRNA bound to the protein. The column was then washed with binding buffer and the proteins were eluted with 20 mM NaH_2_PO_4_, pH 7.5 containing 500 mM NaCl and 650 mM imidazole. The purified proteins were dialyzed overnight against 10 mM Na_2_HPO_4_, 1.75 mM KH_2_PO_4_, pH7.4 containing 137 mM NaCl, 5 mM KCl, 0.5 mM MgCl_2_, and 10% glycerol (PBSM), flash frozen and stored at −80°C.

### RanGTPase protection assay

Recombinant Ran was loaded with [γ-^32^P]GTP as described (5). 1 µM Ran was added to PBSM containing 0.5 nM RanGAP or 0.5 nM RanGAP that had previously been incubated with 150 µM Tween-20 for 30 min in a final volume of 500 µl. Alternatively, 1 µM Ran-[γ-^32^P]GTP was incubated with 150 µM Tween-20 for 30 min prior to being added to PBSM containing 0.5 nM RanGAP. At the specified time, a 50 µl aliquot of the incubation was added to 200 µl of 20 mM phosphoric acid containing 5% activated charcoal (w/v). The charcoal mixtures were then centrifuged for 10 min in 1.5 ml tubes, and 100 µl was removed for scintillation counting to determine the amount of GTP hydrolyzed (5).

### Preparation of HeLa cell lysate and Western blot analysis

HeLa cells were washed with 1× PBS and lifted from the 10 cm dishes by scraping the cells on ice in cold NP-40 lysis buffer (10 mM Tris, pH 7.5, containing 150 mM NaCl, 1 mM Na_3_VO_4_, 10 mM NaF, 2 mM EDTA, and 1% NP-40 (v/v)) containing 1× PIN cocktail and incubated on ice for 10 min with periodic vortexing. The lysates were clarified by centrifugation at 15,000 rpm at 4°C for 10 min. The cell extracts were solubilized in 1× sample buffer and separated by SDS-PAGE on a 10% or 15% polyacrylamide gel. The separated proteins were transferred to ImmobilonP PVDF membranes, and probed with α-NTF2, α-PARP, α-ERK1/2, α-p-ERK1/2, α-p-Akt, or α-p-Ser/Thr/Tyr antibodies (1∶1000) in TBS-T with 0.25% skim milk. The bound antibodies were detected using HRP conjugated α-rabbit and α-mouse secondary antibodies (1∶5000) and the enhanced ECL chemiluminescent detection system.

### Isolation of phosphorylated proteins by chromatography using Phostag-Agarose

PhosTag™-agarose was purchased from Wako Chemicals U. S. A. (Richmond, Virginia). Total cell lysate was prepared from untreated and Tween-20 treated HeLa cells as described above. To determine whether NTF2 is phosphorylated, 75 µg of total cell lysate from untreated or alkaline phosphatase treated samples were incubated with 150 µl of Phostag™ agarose suspension (50% v/v) in the Zn^2+^-bound form for 10 min at room temperature. The resin was centrifuged at 2000 g for 20 sec to remove unbound proteins. The resin was washed three times with Wash buffer (100 mM Tris-CH_3_COOH, 1.0 M CH_3_COONa, pH 7.5) and eluted with three washes of Elution buffer (100 mM Tris-CH_3_COOH, 1.0 M NaCl, 10 mM NaH_2_PO_4_-NaOH, pH 7.5). Eluted protein was precipitated using 25% trichloroacetic acid and the pellet washed once with ice-cold acetone containing 0.05 N HCl and once with ice-cold acetone. The precipitate was dried and resuspended in lysis buffer and subjected to SDS-PAGE on 10% SDS-PAGE gels followed by Western blot analyses.

### Alkaline phosphatase treatment of cell lysates

40 µg of HeLa lysate was incubated with 1 U alkaline phosphatase (Roche) in the presence of 2 mM MgCl_2_ at 37°C for 30 min. Reactions were stopped by the addition of 1× sample buffer and boiling for 5 min. Lysates were then separated by SDS-PAGE and subjected to Western blot analysis.
